# Clinical usefulness of the patient-generated subjective global assessment short form^©^ for nutritional screening in patients with head and neck cancer: a multicentric study

**DOI:** 10.3332/ecancer.2024.1662

**Published:** 2024-02-01

**Authors:** Mariana Duarte Azevedo, Nivaldo Barroso de Pinho, Patrícia de Carvalho Padilha, Livia Costa de Oliveira, Wilza Arantes Ferreira Peres

**Affiliations:** 1 Department of Nutrition and Dietetics, Josué de Castro Institute of Nutrition, Federal University of Rio de Janeiro, Rio de Janeiro, RJ, Brazil; 2 Brazilian Society of Oncology Nutrition, Rio de Janeiro, Brazil; 3 Palliative Care Unit, José Alencar Gomes da Silva National Cancer Institute, Rio de Janeiro, RJ, Brazil

**Keywords:** head and neck cancer, nutritional assessment, malnutrition, PG-SGA SF, diagnostic performance

## Abstract

Nutritional screening and assessment are considered essential steps in nutritional care for cancer patients, malnutrition remains underreported in clinical practice. The aim of this study was to analyse the clinical usefulness of the Patient-Generated Subjective Global Assessment short form (PG-SGA SF©) for nutritional screening in patients with head and neck cancer (HNC). This is a multicentre, cross-sectional study involving patients with HNC. The final score of the PG-SGA SF© was obtained and the nutritional status was diagnosed using the Patient-Generated Subjective Global Assessment (PG-SGA)^®^, classifying them as well-nourished or malnourished. Receiver operating characteristic curve, ordinal logistic regression, and C-statistic were used. In total, 353 patients with HNC were enrolled and the prevalence of malnutrition, according to the PG-SGA^®^, was 64.02% and the median final score of PG-SGA SF© was 11 points. The final score of the PG-SGA SF© had high accuracy (area under the curve = 0.915), and scores ≥9 had the best performance in diagnosing malnutrition. PG-SGA SF© final score ≥9 was associated with malnutrition (odds ratio = 28.32, 95% confidence interval= 15.98–50.17), with excellent discriminatory power (C-statistic = 0.872). In conclusion, the PG-SGA SF© demonstrated excellent performance for nutritional screening in patients with HNC. Given that it is a simple instrument that is faster to administer than the PG-SGA^®^, we recommend its use in clinical practice among such patients.

## Introduction

Cancer-associated malnutrition is related to a combination of reduced food intake and varying degrees of inflammation, leading to changes in body composition and impaired biological function [1]. It may be present at cancer diagnosis and is highly prevalent, varying according to demographic and tumour characteristics [2–5], among other factors, and may be related to the increased risk of occurrence of adverse effects such as reduced survival and quality of life, being recognised as an indicator of a worse prognosis [2, 4, 6–10].

Previous results from the Brazilian survey of oncology nutrition, involving 4,783 patients, showed that those with head and neck cancer (HNC) were at a higher risk of malnutrition (odds ratio (OR) = 3.70, 95% confidence interval (CI) = 2.7–5.2) than those with other primary tumour sites [3]. The most prevalent nutrition impact symptoms among patients with HNC are mucositis, dysphagia, xerostomia, altered taste and smell, and difficulty chewing and swallowing [11, 12], which, associated with the inflammatory response, impact food intake and energy intake while increasing metabolic stress and energy expenditure, resulting in weight loss [13], which may explain the high risk of malnutrition in such cases [3].

The nutritional status must be constantly monitored during treatment, enabling the opportunity for the development of an early individualised nutritional care plan [14]. The patient-generated subjective global assessment (PG-SGA)^®^ is one of the most widely used tools for nutritional risk screening and the diagnosis of malnutrition in cancer patients [15], being recommended by the main international and national societies of nutrition and oncology [14, 16–19]. Its generated subjective global assessment short form (PG-SGA SF^©^) is restricted to the first part of the instrument and includes assessments of history of weight loss, food intake, nutrition impact symptoms and physical function. Jointly, these domains reflect approximately 80%–90% of the total score of the full version. The advantages of the short form are that it can be administered more quickly and that it gives results that are compatible with and related to those given by the full version for the assessment of nutritional risk in some clinical situations [20–22].

Although nutritional screening and assessment are considered essential steps in nutritional care for cancer patients, malnutrition remains underreported in clinical practice [23–25]. To make nutritional monitoring effective, instruments that are practical, accurate, and quick to administer must be made available – qualities that the PG-SGA^©^ can potentially offer. Previous studies have shown that the short version, PG-SGA SF^©^, can be accurate, sensitive, and specific for screening for malnutrition in cancer [20, 23, 26]. However, studies evaluating its performance in nutritional diagnoses of patients with HNC are scarce and have major methodological limitations. Therefore, our objective was to analyse the clinical utility (accuracy, performance, association and discriminatory power) of the PG-SGA SF^©^ for nutritional screening in patients with HNC in a multicenter study.

## Methods

This is a cross-sectional, multicenter study, part of the Brazilian survey of cancer nutrition (BSCN), which evaluated 13.4% of all patients with different types of cancer during treatment admitted to 45 hospitals in different regions of Brazil from August to November 2012. The study was approved by the Research Ethics Committee (no. 34746 of June 22, 2012) and all participants signed an informed consent form. For this proposal, only patients with HNC were considered.

The inclusion criteria for the BSCN were being aged ≥20 years, having a confirmed diagnosis of cancer regardless of location, and consenting to participate in the study, reading and signing the informed consent form. Patients admitted to intensive care, in a coma, or unable to answer the questions that make up the PG-SGA^®^ were excluded. For the present article, only those patients with a primary diagnosis of HNC were considered (all others were excluded). The anatomical sites included in the HNC group were: oral cavity, which comprises oral mucosa, gums, hard palate, tongue, tongue floor; pharynx, which includes oropharynx, nasopharynx, hypopharynx; nasal cavity and paranasal sinuses; glottic and supraglottic larynx and glands.

The following data were retrieved and entered into an electronic database system (http://cpro11505.publiccloud.com.br/) by trained nutritionists: demographic data (age (<60 versus >60 years), gender (male versus female), and region of the country (Central-West versus North-East versus North versus South-East versus South)) and nutritional status (PG-SGA^®^: well-nourished (stage A) versus moderately malnourished or suspected malnutrition (stage B) versus severely malnourished (stage C), and PG-SGA SF^©^ final score (points)) in the first 24 hours of hospitalisation.

The transculturally adapted [27] and validated [28] Brazilian Portuguese version of the PG-SGA^®^ (available at: pt-global.org (^©^FD Ottery, 2005, 2006, 2015)) was used after authorisation was obtained for its use in the research.

The complete PG-SGA^®^ consists of two parts. The first part – the stand-alone shortened version, PG-SGA SF^©^, has four domains: 1 – change in body weight assessed by the percentage of weight loss, with scores varying from 0 to 5; 2 – change in food intake, using the descriptors ‘unchanged’, ‘more than normal’ and ‘less than normal’ (vis-à-vis normal intake), ‘little solid food’, ‘only liquids or nutritional supplements’, ‘very little of anything’, and ‘only tube feeding or only nutrition by vein’, with scores ranging from 0 to 4; 3 – the presence of nutrition impact symptoms in the last 2 weeks, including symptoms that influence food intake, such as constipation, vomiting, dysphagia, pain, among others, scoring up to 24; 4 – and changes in functionality during the previous month, where the patient is characterised as to their capacity to carry out their daily activities, scoring from 0 to 3. The sum of these four items, giving the final score of the PG-SGA SF^©^, can range from 0 to 36 points, with the higher the score, the greater the nutritional risk [21, 22].

The second part of the PG-SGA^®^ includes information regarding 1 – weight loss, 2 – disease and age and their relationship to nutritional needs; 3 – metabolic stress, including fever and steroid use, 4 – and physical examination, including loss/deficit of subcutaneous fat, muscle, and presence of oedema or ascites. After the two parts of the PG-SGA^®^ were administered, patients were subjectively classified as well nourished (stage A), moderately malnourished or suspected malnutrition (stage B), or severely malnourished (stage C). To assess the reliability of the nutritionists’ classification using the PG-SGA^®^, three hospitals participating in the BSCN were randomly selected over the period of data collection. In these places, nutritionists participating in the research and experienced researchers administered the PG-SGA^®^ to the same patients independently, for further statistical comparison.

### Statistical analysis

Reliability between selected nutritionists and experienced researchers was determined using Cohen’s *κ*, which is based on the number of concordant responses between the different raters, using SAS^®^, version 6.11 (SAS Institute Inc). The coefficient was interpreted as proposed by Shrout [29], with agreement >80% signifying substantial, 61%–80% moderate, 41%–60% fair, 10%–40% slight, and <10% virtually none. The reliability of the patients’ nutritional status classification by nutritionists using the PG-SGA^®^ was considered substantial, with 90% agreement (CI: 41%–100%).

The other analyses were performed using STATA 13.1 (Stata Corp., College Station, Texas, USA). Statistical significance was set at *p*-value <0.05. Conformity to the normal curve was verified in order to assess the symmetry of the variables and the Kolmogorov-Smirnov test was used to test the normality of the sample distribution. The numerical variables were not normally distributed and were presented as medians and interquartile ranges (IQR, 25th and 75th percentiles), while the categorical variables were presented as frequencies (*n*) and percentages (%).

The receiver operating characteristic (ROC) curve was used to identify the area under the curve (AUC) to assess the accuracy of the global score of the PG-SGA SF^©^ in relation to the diagnosis of malnutrition (sum of stages B and C). AUC ≥0.700 was considered statistically significant [30]. In addition, the diagnostic performance of different cutoff points of the global score of the PG-SGA SF^©^ was evaluated by determining their sensitivity, specificity, and positive and negative likelihood ratio. Sensitivity is the ability of the method to correctly identify true positives (malnourishment, i.e., stages B and C). Specificity is the ability to identify true negatives (well nourished, i.e., stage A). We selected a cutoff point with the highest sensitivity and specificity, prioritising the one with the highest sensitivity (percentage of malnourished people correctly identified as such), which is what is necessary to obtain good discrimination in a nutritional assessment tool.

To assess the association between the selected PG-SGA SF^©^ cutoff point and the diagnosis of malnutrition (stage B or C, as a polytomous outcome indicative of severity), univariate and multivariate ordinal logistic regression analyses were used, taking as measures of effect the OR with 95% CI. The variables selected for the adjustment of the multiple model were: age, sex and geographical region of the country.

In addition, the C-statistic was used to assess the discrimination of the PG-SGA SF^©^ to predict malnutrition. A C-statistic of 0.5 indicates that the model predicts the outcome as well as chance (i.e., equal numbers of true and false positives), 0.7 to <0.8 indicates acceptable discrimination, 0.8 to <0.9 indicates excellent discrimination, 0.9 to <1.0 is outstanding discrimination, and 1.0 is a perfect prediction [31].

To check our sampling power, we performed a *post hoc* test using the online tool available at https://clincalc.com/Stats/Power.aspx, considering dichotomous results (prevalence of malnutrition: yes versus no) and an alpha error of 0.05. With the number of patients with HNC included in this article, the sampling power was 100%.

## Results

**Figure 1.**
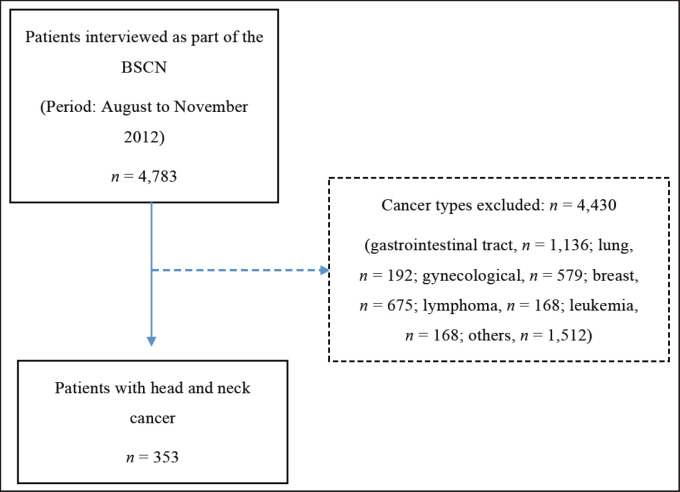
Flowchart of selection of patients with HNC evaluated in the BSCN. n = number of observations; BSCN = Brazilian survey of cancer nutrition.

A total of 353 patients with HNC were included (Figure 1), mostly male (79.32%) and aged ≥60 years (61.11%). Regarding demographic distribution, the patients were mainly from the South-East (48.20%) and North-East (26.06%) regions. The majority (55.24%) had 1–3 nutrition impact symptoms, the most prevalent of which were dysphagia (40.16%) and poor appetite (22.90%). The prevalence of malnutrition, according to the PG-SGA^®^, was 64.02% (*n* = 226) and the median PG-SGA SF^©^ final score was 11 (IQR: 5–17) points (Table 1).

**Figure 2.**
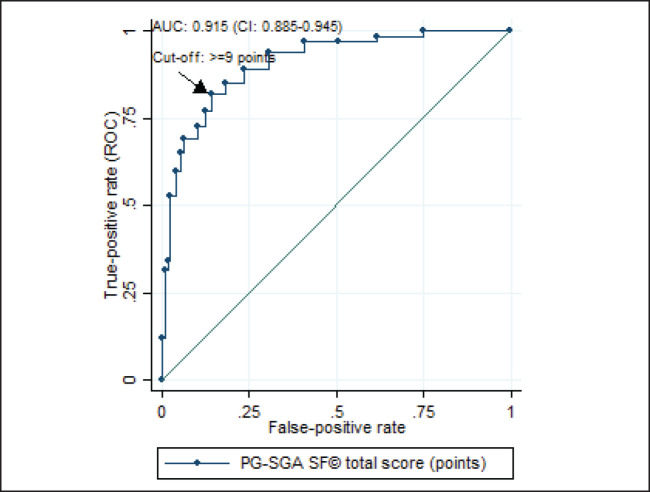
ROC curve of the final score of the PG-SGA SF^©^ in relation to the diagnosis of malnutrition* in patients with HNC evaluated in the BSCN (n = 353). ROC = Receiver operating characteristic; AUC = area under the curve; CI = confidence interval; PG-SGA SF^©^ = Patient-generated subjective global assessment short form. *malnutrition diagnosed using the patient-generated subjective global assessment^®^ (sum of stages B and C).

According to the analysis of the ROC curve, the accuracy of the PG-SGA SF^©^ final score was excellent (AUC = 0.915, 95% CI = 0.885–0.945) for the diagnosis of malnutrition (Figure 2), with ≥9 as the cutoff point giving the best performance for identifying malnourished patients (sensitivity = 84.96%, specificity = 85.83%) (Table 2).

The multivariate polytomous ordinal logistic regression demonstrated that the PG-SGA SF^©^ final score ≥9 (OR = 28.32, 95% CI = 15.98–50.17) was associated to malnutrition (moderate or severe), regardless of age, sex, or region of the country. In addition, the short form showed excellent discriminatory power according to the C-statistic (0.872) (Table 3).

## Discussion

In order to analyse the clinical usefulness of the PG-SGA SF^©^ in nutritional screening in patients with HNC, our data analysis demonstrates that it presented excellent accuracy, with a global score of ≥9 being the cutoff point that had the best performance, with independent association with malnutrition and excellent discriminatory power. Therefore, its use could be recommended in clinical practice for nutritional screening and diagnosis for HNC patients.

It is important to highlight that, in line with national and international evidence [6, 8, 32–34], our results showed higher frequencies of HNC among men and older people. In addition, the highest rates of this type of cancer were found in the South-East (48.20%), followed by the North-East (26.06%), corroborating what is reported in the national literature and reflecting the distinct characteristics of Brazil’s geographic regions [34].

Most of the patients with HNC had 1–3 nutrition impact symptoms (55.24%), the most prevalent of which were dysphagia (40.16%) and hyporexia (22.90%). It is known that HNC affects structures that are critical for basic functions, such as breathing, swallowing, and verbal communication. Complications in these areas resulting from the disease and its treatment can lead to severe damage and physiological changes that cause difficulties in chewing, dysphagia, xerostomia, trismus, salivary problems, taste alteration, mucositis and pain in the oral cavity, impacting negatively on nutritional status [13, 35].

Our results confirm the high prevalence of malnutrition in this group, in line with the literature [3, 6, 33, 36]. Neoh *et al* [36] evaluated nutritional changes during radiotherapy, with or without chemotherapy, in 50 patients with HNC. Using the PG-SGA^®^, they found an increase in the prevalence of malnutrition from 56% pre-treatment to 100% post-treatment, with an average unintentional loss of body weight of 4.53 (±0.41) kg at the end of radiotherapy. Nutritional parameters such as muscle mass, fat mass, body mass index, dietary energy and protein intake decreased significantly (*p* < 0.0001), while the prevalence of symptoms (mainly taste alteration and dry mouth) and oral supplement intake (which reduced weight loss in those with adequate supplement intake (*p* = 0.013)) increased significantly (*p* < 0.0001).

Despite its high magnitude and recognised deleterious effects on clinical outcomes [2, 4, 6–10], malnutrition remains underreported in clinical practice [24, 25] and, when recognised, may not be properly treated, contrary to recommendations that nutritional status be taken into account from the moment of cancer diagnosis, for early identification and treatment of its probable alterations [19].

Considering that the assessment of nutritional status is a dynamic, systematic process, consisting of data collection and interpretation to support decision-making, regardless of the stage of the disease [14], and that there are barriers to carrying out nutritional assessments in clinical practice, there is a clear need for simple and accurate tools such as the PG-SGA SF^©^. We found that the final score of the PG-SGA SF^©^ had excellent accuracy (AUC = 0.915, 95% CI = 0.885–0.945), with scores ≥9 having the best performance for the diagnosis of malnutrition (sensitivity = 85.0%, specificity = 85.8%). Such measures demonstrate the good capacity of this instrument to correctly identify true positives (malnourished patients) and true negatives (well-nourished patients).

Although previous studies have evaluated the good performance of the PG-SGA SF^©^ for the diagnosis of malnutrition, most of them involved patients with other tumour types [20, 26]. There are few studies based on patients with HNC, and those that exist have major methodological limitations. Jager-Wittenaar *et al* [23] found that the PG-SGA SF^©^ had better accuracy for diagnosing malnutrition when compared to the Malnutrition Universal Screening Tool, with high sensitivity (73%) and specificity (100%) and the ability to predict both adverse effects and the improvement of clinical outcomes. However, the sample size was limited (*n* = 78) and the study was conducted at a single centre. In a cross-sectional study involving 355 patients with different types of cancer undergoing chemotherapy, Carriço *et al* [20] found that the PG-SGA SF^©^ was adequate for identifying the nutritional risk of patients, comparing its results with those given by the PG-SGA^®^. According to the PG-SGA SF^©^, the majority (69.3%, *n* = 246) of the participants had at least one risk factor for malnutrition and according to the PG-SGA^®^, 50% (*n* = 177) presented nutritional risk or actual malnutrition (stages B and C). The agreement of the results was moderate (*k* coefficient = 0.62, *p* < 0.001), with the PG-SGA SF^©^ having good sensitivity (95%) and specificity (67%) for identifying nutritional risk and malnutrition.

In addition to evaluating PG-SGA SF^©^ for its sensitivity and specificity for predicting nutritional status, studies have shown that it covers all the domains recommended by ESPEN and ASPEN for defining malnutrition, demonstrating its content validity [37]. Although the nutritional screening and diagnosis instruments used in clinical practice in hospitals are already based mainly on criteria that reflect the characteristics of malnutrition, to combat malnutrition at an early stage it may be valuable to additionally identify its risk factors, which is where PG-SGA SF^©^ could help [38].

Regardless of age, sex, or region of the country, a PG-SGA SF^©^ final score ≥9 points (OR: 29.48, 95% CI: 16.57–52.44) was associated with malnutrition (moderate or severe), with excellent predictive accuracy (C-statistic = 0.872). This indicates that care protocols could be based on its results, offering a speedier nutritional diagnosis, and enabling an earlier onset of actions to combat malnutrition. Drawing on data from 1,157 patients with HNC, Martin *et al* [8] found that among those with the same final score (PG-SGA SF^©^ ≥9 points), the prevalence of sarcopenia was 50% and the prevalence of myosteatosis was 49%.

Finally, it is important to highlight the results of the study developed by Jager-Wittenaar *et al* [32] with 59 patients with HNC to assess the feasibility of self-completion of the PG-SGA SF^©^ and awareness of the risk of malnutrition after self-reporting. The average time required to complete the PG-SGA SF^©^ was 2 minutes and 41 seconds (IQR: 1 minute 49 seconds – 3 minutes 50 seconds). Forty-eight percent of the patients needed help with completion, indicating acceptable feasibility. For 7/9 questions about awareness of the risk of malnutrition, >50% of the participants responded positively, showing that awareness may increase after the patient completes the questionnaire [32].

Some limitations of our study should be highlighted, such as the fact that we only included inpatients, making it likely inappropriate to apply our conclusions to an outpatient population. Inpatient-only inclusion could probably overestimate malnutrition. In addition, another limitation was the fact that the PG-SGA^®^ was not filled out by the patient, but by different nutritionists, which may have interfered with the results since the professionals know how to fill in the instrument more clearly than the patients [32]. However, these professionals were previously trained, and this method of completion is probably the best way to minimize the overestimation of symptoms [21], in addition to the fact that the reliability of the nutritional status classification by nutritionists was considered good. Another limitation is that the data on dietary habits (which would be an important variable in this context) were not collected it was not possible to add it to the statistical analysis. Despite the data analysed having been collected more than 10 years ago, it should be noted that they were obtained in a large multicenter study involving different hospitals across the country, reinforcing the relevance of the finding.

## Conclusion

The PG-SGA SF^©^ had an excellent performance in nutritional screening in patients with HNC. Given that it is a simple instrument that is faster to administer than the full version, we recommend its use in clinical practice. Its use in care protocols could enable a speedier diagnosis of malnutrition, helping to combat the underreporting of malnutrition and enabling faster decision-making regarding nutritional intervention, which may contribute to the optimisation of resources and referral to nutritional rehabilitation for those who really need it. However, more studies need to be developed in patients with other tumour types, and with the aim of comparison of the tools (PG-SGA SF^©^ versus complete PG-SGA^©^).

## Conflicts of interest

The authors declare that they have no conflict of interest.

## Funding

This research did not receive any specific grant from funding agencies in the public, commercial, or not-for-profit sectors.

## Figures and Tables

**Table 1.** Demographic and nutritional characteristics of patients with HNC assessed in the Brazilian survey of oncology nutrition (*n* = 353).

Variables	*N*	%
Sex		
Female	73	20.68
Male	280	79.32
Age (years)		
<60	138	38.89
≥60	215	61.11
Region of country		
Central-West	43	12.18
North-East	92	26.06
North	7	1.98
South-East	170	48.16
South	41	11.61
Number of nutrition impact symptoms		
0	102	28.90
1–3	195	55.24
≥3	56	15.86
Most prevalent nutrition impact symptoms		
Dysphagia	142	40.23
Poor appetite	81	22.95
Xerostomia	69	19.55
Mouth pain	63	17.85
PG-SGA SF© final score (points) (median/IQR)	11	5-17
PG-SGA SF© final score (points)		
0–1	32	9.07
2–3	38	10.76
4–8	80	22.66
≥ 9	203	57.51
Nutritional diagnosis by PG-SGA®		
Well nourished (stage A)	127	35.98
Moderately malnourished or suspected malnutrition (stage B)	140	39.66
Severely malnourished (stage C)	86	24.36

*n* = number of observations; % = frequency; IQR = interquartile range (25th to 75th percentile); PG-SGA SF^©^ = Patient-Generated Subjective Global Assessment short form; PG-SGA^®^ = Patient-Generated Subjective Global Assessment

**Table 2.** Performance measures of the cut-off points of the final score of the PG-SGA SF© in relation to the diagnosis of malnutrition^a^ in patients with HNC evaluated in the BSCN (*n* = 353).

Performance measures	PG-SGA SF^©^ final score (points)							
	2	3	4	5	6	7	8	9^b^
Sensitivity	100%	98.23%	96.90%	96.90%	93.81%	88.94%	84.96%	84.96%
Specificity	25.20%	38.58%	49.61%	59.06%	69.29%	76.38%	81.89%	85.83%
LR+	1.34	1.60	1.92	2.37	3.05	3.76	4.69	5.77
LR-	0	0.04	0.06	0.05	0.08	0.14	0.18	0.21

a malnutrition diagnosed using the patient-generated subjective global assessment^®^ (sum of stages B and C)

b cutoff points above nine had lower sensitivity (and decreasing) values

*n* = number of observations; LR = likelihood ratio; PG-SGA SF^©^ = Patient-Generated Subjective Global Assessment short form

**Table 3.** Ordinal logistic regression of PG-SGA SF© final score >9 points in relation to the diagnosis of malnutrition^a^ in patients with HNC evaluated in the BSCN (*n* = 353).

Variables	Univariate		Multivariate		C-statistic
	OR (95% CI)	*p*-value	OR (95% CI)	*p*-value	
**PG-SGA SF** ^©^ ** final score >9 points**	25.40 (14.55–44.33)	**<0.001**	28.32 (15.98–50.17)	**<0.001**	0.872
Adjusting factors					
Age >60 years	0.23 (0.03–1.80)	0.161	0.92 (0.57–1.50)	0.751	-
Sex male	1.96 (1.20–3.21)	**0.007**	2.90 (1.60–5.25)	**<0.001**	-
Region South-East	1.12 (1.31–2.84)	**0.009**	1.92 (0.99–3.41)	0.060	-

a malnutrition diagnosed using the patient-generated subjective global assessment^®^ (stages B or C, as polytomous outcome indicative of severity)

*n* = number of observations; OR = odds ratio; CI = confidence interval; PG-SGA SF^©^ = Patient-Generated Subjective Global Assessment short form

## References

[1] Cederholm T, Barazzoni R, Austin P (2017). ESPEN guidelines on definitions and terminology of clinical nutrition. Clin Nutr.

[2] Oliveira LC, Abreu GT, Lima LC (2020). Quality of life and its relation with nutritional status in patients with incurable cancer in palliative care. Support Care Cancer.

[3] Pinho NB, Martucci RB, Rodrigues VD (2019). Malnutrition associated with nutrition impact symptoms and localization of the disease: results of a multicentric research on oncological nutrition. Clin Nutr.

[4] Cunha MS, Wiegert EVM, Calixto-Lima L (2018). Relationship of nutritional status and inflammation with survival in patients with advanced cancer in palliative care. Nutrition.

[5] Muscaritoli M, Lucia S, Farcomeni A (2017). Prevalence of malnutrition in patients at first medical oncology visit: the PreMiO study. Oncotarget.

[6] Santos A, Santos IC, Reis PF (2021). Impact of nutritional status on survival in head and neck cancer patients after total laryngectomy. Nutr Cancer.

[7] Santos IM, Mendes L, Carolino E (2020). Nutritional status, functional status, and quality of life – what is the impact and relationship on cancer patients?. Nutr Cancer.

[8] Martin L, Gioulbasanis I, Senesse P (2020). Cancer-associated malnutrition and CT-defined sarcopenia and myosteatosis are endemic in overweight and obese patients. JPEN J Parenter Enteral Nutr.

[9] Cong M, Zhu W, Wang C (2020). Nutritional status and survival of 8247 cancer patients with or without diabetes mellitus– results from a prospective cohort study. Cancer Med.

[10] Maia FCP, Silva TA, Vasconcelos Generoso S (2020). Malnutrition is associated with poor health-related quality of life in surgical patients with gastrointestinal cancer. Nutrition.

[11] Langius JAE, Doornaert P, Spreeuwenberg MD (2010). Radiotherapy on the neck nodes predicts severe weight loss in patients with early stage laryngeal cancer. Radiother Oncol.

[12] Ravasco P, Monteiro-Grillo I, Marques Vidal P (2005). Impact of nutrition on outcome: a prospective randomized controlled trial in patients with head and neck cancer undergoing radiotherapy. Head Neck.

[13] Farhangfar A, Makarewicz M, Ghosh S (2014). Nutrition impact symptoms in a population cohort of head and neck cancer patients: multivariate regression analysis of symptoms on oral intake, weight loss and survival. Oral Oncol.

[14] Arends J, Baracos V, Bertz H (2017). ESPEN expert group recommendations for action against cancer-related malnutrition. Clin Nutr.

[15] Jager-Wittenaar H, Ottery FD (2017). Assessing nutritional status in cancer: role of the patient-generated subjective global assessment. Curr Opin Clin Nutr Metab Care.

[16] Instituto Nacional de Câncer José de Alencar Gomes da Silva (2016). Consenso nacional de nutrição oncológica.

[17] Horie LM, Barrére APN, Castro MG (2019). Diretriz BRASPEN de terapia nutricional no paciente com câncer. Braz Soc Parenter Enteral Nutr.

[18] Arends J, Strasser F, Gonella S (2021). Cancer cachexia in adult patients: ESMO clinical practice guidelines. ESMO Open.

[19] Muscaritoli M, Arends J, Bachmann P (2021). ESPEN practical guideline: clinical nutrition in cancer. Clin Nutr.

[20] Carriço M, Guerreiro CS, Parreira A (2021). The validity of the patient-generated subjective global assessment Short-form^©^ in cancer patients undergoing chemotherapy. Clin Nutr ESPEN.

[21] Abbott J, Teleni L, McKavanagh D (2016). Patient-generated subjective global assessment short form (PG-SGA SF) is a valid screening tool in chemotherapy outpatients. Support Care Cancer.

[22] Vigano AL, Tomasso J, Kilgour RD (2014). The abridged patient-generated subjective global assessment is a useful tool for early detection and characterization of cancer cachexia. J Acad Nutr Diet.

[23] Jager-Wittenaar H, Ottery F, Bats H (2016). Diagnostic accuracy of PG-SGA SF, MUST and SNAQ in patients with head and neck cancer. Clin Nutr.

[24] Durán-Poveda M, Jimenez-Fonseca P, Sirvent-Ochando M (2018). Integral nutritional approach to the care of cancer patients: results from a Delphi panel. Clin Transl Oncol.

[25] Müller-Richter U, Betz C, Hartmann S (2017). Nutrition management for head and neck cancer patients improves clinical outcome and survival. Nutr Res.

[26] De Groot LM, Lee G, Ackerie A (2020). Malnutrition screening and assessment in the cancer care ambulatory setting: mortality predictability and validity of the patient-generated subjective global assessment short form (PG-SGA SF) and the GLIM criteria. Nutrients.

[27] Campos JADB, Prado CD (2012). Cross-cultural adaptation of the Portuguese version of the patient-generated subjective global assessment. Nutr Hosp.

[28] Gonzalez MC, Borges LR, Silveira DH (2010). Validação da versão em português da avaliação subjetiva global produzida pelo paciente n.d.:7. Validation of a Portuguese version of patient-generated subjective global assessment. Rev Bras Nutr Clin.

[29] Shrout PE (1998). Measurement reliability and agreement in psychiatry. Stat Methods Med Res.

[30] Metz CE (1978). Basic principles of ROC analysis. Semin Nucl Med.

[31] Hosmer DW, Lemeshow S (2000). Applied Logistic Regression.

[32] Jager-Wittenaar H, Bats HF, Welink-Lamberts BJ (2020). Self-completion of the patient-generated subjective global assessment short form is feasible and is associated with increased awareness on malnutrition risk in patients with head and neck cancer. Nutr Clin Pract.

[33] Yanni A, Dequanter D, Lechien JR (2019). Malnutrition in head and neck cancer patients: Impacts and indications of a prophylactic percutaneous endoscopic gastrostomy. Eur Ann Otorhinolaryngol Head Neck Dis.

[34] Instituto Nacional de Câncer José Alencar Gomes da Silva (2019). Estimativa 2020: incidência de câncer no Brasil.

[35] Crowder SL, Douglas KG, Yanina Pepino M (2018). Nutrition impact symptoms and associated outcomes in post-chemoradiotherapy head and neck cancer survivors: a systematic review. J Cancer Surviv.

[36] Neoh MK, Abu Zaid Z, Daud ZAM (2020). Changes in nutrition impact symptoms, nutritional and functional status during head and neck cancer treatment. Nutrients.

[37] Balstad TR, Bye A, Jenssen CR (2019). Patient interpretation of the patient-generated subjective global assessment (PG-SGA) short form. Patient Prefer Adherence.

[38] Dewansingh P, Euwes M, Krijnen WP (2021). Patient-generated subjective global assessment short form better predicts length of stay than short nutritional assessment questionnaire. Nutrition.

